# Risk factors for unplanned cesarean delivery among pregnant individuals with pre‐existing diabetes

**DOI:** 10.1002/pmf2.70303

**Published:** 2026-05-20

**Authors:** Marie‐Julie Trahan, Qi Yan, Meghan Angley, Kim A. Boggess, Jerrie S. Refuerzo, Kacey Y. Eichelberger, Gladys A. Ramos, Celeste Durnwald, Kjersti M. Aagaard, Kedra Wallace, Christina Scifres, Todd Rosen, Sherri Longo, Noelia Zork

**Affiliations:** ^1^ Department of Obstetrics & Gynecology, Division of Maternal‐Fetal Medicine Columbia University Irving Medical Center New York New York USA; ^2^ Department of Obstetrics & Gynecology—Biostatistics Columbia University New York New York USA; ^3^ Department of Obstetrics and Gynecology University of North Carolina at Chapel Hill School of Medicine Chapel Hill North Carolina USA; ^4^ Department of Obstetrics and Gynecology University of Texas Health Houston McGovern Medical School Houston Texas USA; ^5^ Department of Obstetrics and Gynecology, Division of Maternal‐Fetal Medicine, Prisma Health University of South Carolina School of Medicine Greenville Greenville South Carolina USA; ^6^ Department of Obstetrics, Gynecology and Reproductive Sciences University of California San Diego, La Jolla California USA; ^7^ Department of Obstetrics and Gynecology, Division of Maternal Fetal Medicine University of Pennsylvania Philadelphia Pennsylvania USA; ^8^ HCA Healthcare and HCA Research Institute Nashville Tennessee USA; ^9^ Fetal Care and Surgery Center (FCSC), Division of Fetal Medicine and Surgery Boston Children's Hospital, Harvard Medical School Boston Massachusetts USA; ^10^ Department of Pharmacology and Toxicology University of Mississippi Medical Center Jackson Mississippi USA; ^11^ Department of Obstetrics and Gynecology Indiana University School of Medicine Indianapolis Indiana USA; ^12^ Department of Obstetrics and Gynecology Rutgers Health/Robert Wood Johnson Medical School New Brunswick New Jersey USA; ^13^ Maternal and Fetal Medicine Ochsner Health New Orleans Louisiana USA

**Keywords:** cesarean delivery, HbA1c, labor, pregestational diabetes, pregnancy, Type 2 diabetes, vaginal delivery

## Abstract

**Background:**

Diabetes in pregnancy is associated with an increased risk of cesarean delivery, including unplanned or emergency cesarean delivery. It is unclear which risk factors confer increased risk of cesarean among pregnancies with Type 2 diabetes or early gestational diabetes. Given the increased maternal risks associated with cesarean delivery, a better understanding of risk factors for unplanned cesarean delivery may improve patient care and reduce delivery complications among this high‐risk population.

**Objective:**

The objective of this study was to identify risk factors for unplanned primary cesarean delivery among pregnant individuals with Type 2 diabetes or diabetes diagnosed early in pregnancy.

**Study design:**

This was a secondary analysis of the Medical Optimization and Management of Pregnancies with Overt Type 2 Diabetes (MOMPOD) randomized controlled trial. Participants with pre‐existing Type 2 diabetes or diabetes identified prior to 23 weeks were included if they had a trial of labor. Baseline and pregnancy characteristics were compared between those with a vaginal delivery and those with an unplanned cesarean delivery using descriptive and logistic regression analyses, controlling for potential confounders.

**Results:**

Of 794 enrolled participants, 405 met prespecified inclusion criteria for this secondary analysis; of these, 144 (36%) had an unplanned cesarean delivery and 261 (64%) had a vaginal delivery. Indications for cesarean delivery were failed induction of labor (65/144; 45%), fetal intolerance to labor (51/144; 35%), and cephalopelvic disproportion or failure to progress (34/144; 24%). Compared to those with a vaginal delivery, participants with an unplanned cesarean delivery had greater median gestational weight gain (11.6 kg [interquartile range, IQR 6.8–15.4] vs. 10 kg [IQR, 4.4–14.9], *p* = 0.019), enrollment HbA1c ≥ 6.5% (69% vs. 56%, *p* = 0.0239), third trimester HbA1c ≥ 6.5% (39% vs. 25%, *p* = 0.0459), and higher median glucose levels in labor (130.0 mg/dL [IQR 112.0–161.5] vs. 121.0 mg/dL [IQR 100.0–144.0], *p* = 0.0025). There were no differences in the timing of diabetes diagnosis, metformin use, or birthweight between groups. In multivariable adjusted analysis controlling for nulliparity, Hispanic origin, marital status, pre‐existing hypertension requiring medication, preeclampsia, and cigarette use in pregnancy, enrollment HbA1c (mean gestational age 11.2 weeks) (median HbA1c: 7.3% for CD vs. 6.85% for vaginal delivery; aOR 1.27 [95% confidence interval, CI 1.10, 1.46]), HbA1c ≥ 6.5% at enrollment (69% for cesarean delivery vs. 56% for vaginal delivery; aOR 2.26 [95% CI 1.28, 3.98]), HbA1c ≥ 6.5% in the third trimester (39% for cesarean delivery vs. 25% for vaginal delivery; aOR 2.78 [95% CI 1.21, 6.39]), and induction (88% for cesarean delivery vs. 76% for vaginal delivery; aOR 2.12 [95% CI 1.08, 4.17]) were associated with unplanned cesarean delivery. There was no difference in gestational weight gain or the highest glucose level in labor between groups.

**Conclusions:**

In patients with Type 2 diabetes or diabetes diagnosed in early pregnancy who are attempting vaginal delivery, early pregnancy HbA1c, third trimester HbA1c, and induction of labor are associated with an increased risk of unplanned primary cesarean delivery. Preconception counseling should focus on improved glycemic management prior to and in early pregnancy as a potential strategy to reduce the risk of unplanned cesarean delivery for pregnant individuals with pre‐existing diabetes or diabetes diagnosed in early pregnancy.

## 1 INTRODUCTION

In the United States, pregestational diabetes complicates 1%–2% of pregnancies [1]. The incidence of Type 2 diabetes (T2D), the most common form of pregestational diabetes, is rising and continues to disproportionately affect Black, Indigenous, Hispanic, and other people of color [1, 2, 3, 4]. Important contributors to the rise in T2D among pregnant individuals include elevated body mass index (BMI), physical inactivity, suboptimal diet, and advancing maternal age, among others [2].

Diabetes in pregnancy is associated with maternal, fetal, and neonatal complications [1, 5]. Pregnant individuals with pregestational diabetes or gestational diabetes (GDM) have a greater risk of requiring a cesarean delivery (CD) compared to those without diabetes [1, 4, 6, 7]. In particular, compared to patients without diabetes, pregnant patients with diabetes face a two‐ to threefold increased risk of unplanned or emergency CD while attempting a vaginal delivery (VD) [6, 8], which is associated with higher rates of surgical complications compared to planned CD, including postpartum hemorrhage, infection, wound dehiscence, and need for postpartum reoperation. These complications contribute to maternal morbidity and may have implications for future pregnancies [9, 10].

Prior studies have identified nulliparity, obesity, gestational weight gain, insulin use, and an ultrasound diagnosis of a large‐for‐gestational age fetus as risk factors for CD among pregnant individuals with GDM diagnosed in the third trimester [5, 11]. It is unclear whether these same risk factors apply to pregnant individuals with pre‐existing or early pregnancy diabetes, or how glucose control prior to and in early pregnancy affects the risk of unplanned or emergency CD. Given the maternal risks associated with CD, a better understanding of risk factors for unplanned CD may improve patient care and reduce delivery complications among this high‐risk obstetrical population.

The objective of this study was to evaluate clinical risk factors for unplanned primary CD among pregnant individuals with T2D and early GDM.

## 2 MATERIALS AND METHODS

### 2.1 Study design and population

This was a secondary analysis of the Medical Optimization and Management of Pregnancies with Overt Type 2 Diabetes (MOMPOD) randomized controlled trial (RCT).

**FIGURE 1:**
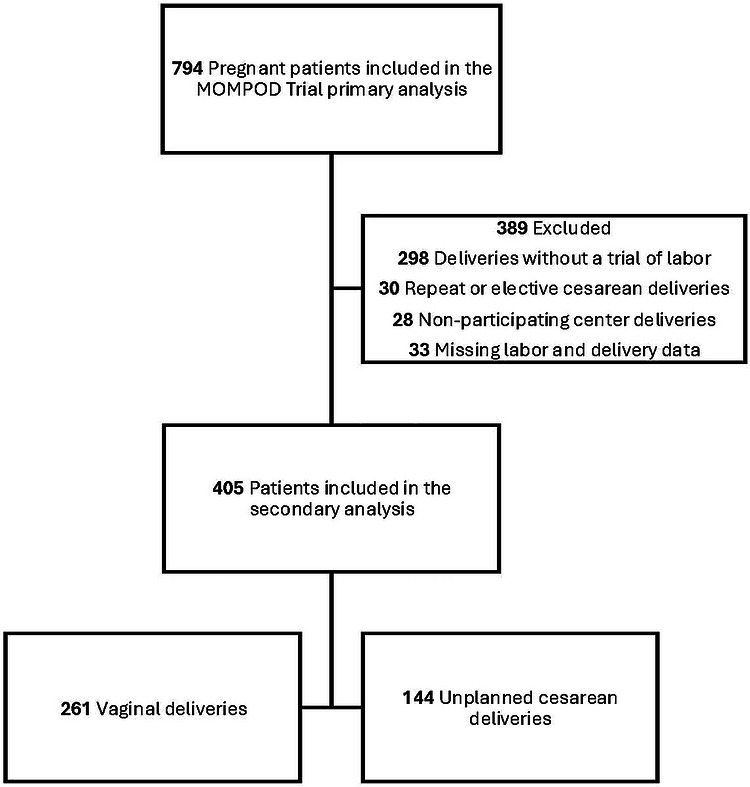
Study population flowchart. Elective cesarean delivery (CD) refers to primary CD in the absence of labor. MOMPOD, Medical Optimization and Management of Pregnancies with Overt Type 2 Diabetes.

For MOMPOD, adults between the ages of 18 and 45 years from 17 US participating centers with a singleton pregnancy and either pre‐existing T2D or diabetes diagnosed early in gestation were enrolled between 2017 and 2021 [12]. Enrollment occurred prior to 23 weeks of gestation, with the majority of patients enrolled prior to 18 weeks of gestation [12]. Early GDM was defined as diabetes diagnosed prior to 23 weeks of gestation [12]. Early GDM was diagnosed based on either ≥ 1 abnormal value on a 75‐g oral glucose tolerance test (OGTT), ≥ 2 abnormal values on a 100‐g OGTT after a positive 50‐g OGTT, HbA1c ≥6.5%, fasting capillary blood glucose level > 126 mg/dL, or a random capillary blood glucose level ≥ 200 mg/dL [12]. MOMPOD participants were all treated with insulin and randomized to add either metformin or placebo [12]. For this analysis, MOMPOD participants with T2D or early GDM were included if they had a trial of labor. MOMPOD participants were excluded if they had a prior CD, had a primary CD in the absence of labor, or if they delivered at a non‐participating center (Figure 1). Participants were then divided into two groups based on mode of delivery: (1) VD and (2) unplanned CD. We did not restrict participants based on gestational age at delivery.

### 2.2 Participant characteristics

Baseline characteristics evaluated included maternal age, race, ethnicity, marital status, primary source of insurance, pre‐pregnancy BMI, timing of diabetes diagnosis (T2D diagnosed prior to pregnancy or early GDM based on MOMPOD definitions), pre‐existing hypertension requiring medication, cigarette use, and parity. Pregnancy characteristics evaluated included gestational age (GA) at delivery, type of labor (spontaneous or induced), and neonatal birthweight. Diabetes characteristics evaluated included hemoglobin A1c (HbA1c) at enrollment, HbA1c in the third trimester, gestational weight gain, and the highest glucose level in labor.

### 2.3 Statistical methods

Baseline, pregnancy, and diabetes characteristics were compared between those with a VD and those with an unplanned CD using descriptive and logistic regression analyses. Logistic regression was performed to test the association between unplanned CD and six exposures (HbA1c at enrollment [continuous and ≥ 6.5%], HbA1c in the third trimester ≥ 6.5%, gestational weight gain, induction of labor, and the highest glucose level in labor) with adjustment for potential confounders, including nulliparity, Hispanic origin, marital status, pre‐existing hypertension requiring medication, preeclampsia, and cigarette use in pregnancy. Characteristics were considered potential confounders if they were significantly different between CD and VD groups, potentially associated with the outcome, and not an intermediate in the causal pathway between the exposures and unplanned CD. Crude and adjusted odds ratios (aOR) with 95% confidence intervals (CI) were calculated. A posteriori, generalized additive models were used to determine the relationship between 1% change in enrollment HbA1c and CD, adjusting for potential confounders. *p* values < 0.05 were considered statistically significant. Statistical analyses were performed using R Software (Version 4.4.1).

## 3 RESULTS

Of the 794 participants enrolled and included in the MOMPOD study, 405 (51%) participants met prespecified inclusion criteria for this secondary analysis and attempted a trial of labor (Figure 1). Among these, 144 (36%) had an unplanned CD and 261 (64%) had a VD (Figure 1). For participants who had an unplanned CD, the most common specified indications for CD were as anticipated in this population, including failed induction of labor (65/144; 45%), fetal intolerance to labor (51/144; 35%), and cephalopelvic disproportion or failure to progress (34/144; 24%).

There was no difference in the age, race, insurance type, or pre‐pregnancy BMI between groups; however, there were a greater number of VD in participants of Hispanic origin (59.0% vs. 47.2%) and those who were married or living with a partner (73.2% vs. 61.8%) (Table 1). There were more nulliparas and a higher prevalence of labor inductions, hypertension requiring medication, cigarette use, and preeclampsia in the unplanned CD group.

**TABLE 1:** Baseline, diabetes, and pregnancy characteristics by mode of delivery.

	Unplanned CD (*n* = 144)	VD (*n* = 261)	*p* value
Baseline characteristics			
Maternal age (years) (mean, SD)	32.0 ± 6.0	33.0 ± 5.7	0.100
Race (*n*, %)			0.264
White	52 (36.1)	93 (35.6)	
Black	51 (35.4)	73 (28.0)	
Asian	8 (5.6)	11 (4.2)	
American Indian or Alaska native	1 (0.7)	1 (0.4)	
Multi‐race	2 (1.4)	3 (1.2)	
Not reported or declined to report	30 (20.8)	80 (30.7)	
Hispanic origin (*n*, %)	68 (47.2)	154 (59.0)	0.023
Married or living with partner (*n*, %)	89 (61.8)	191 (73.2)	0.018
Primary source of insurance (*n*, %)			0.294
Private	28 (19.4)	50 (19.3)	
Military	2 (1.4)	1 (0.4)	
Medicaid, federal, or state insurance	107 (74.3)	185 (71.4)	
None	7 (4.9)	23 (8.9)	
Pre‐pregnancy BMI (kg/m^2^) (median, IQR)	34.5 (30.4–40.9)	33.5 (28.9–39.9)	0.088
Pre‐existing hypertension requiring medication (*n*, %)	41 (28.5)	45 (17.4)	0.009
Preeclampsia (*n*, %)	60 (41.7)	76 (29.1)	0.011
Cigarette use in pregnancy (*n*, %)	17 (11.8)	12 (4.6)	0.008
Diabetes characteristics			
Timing of diabetes diagnosis (*n*, %)			0.068
Pre‐existing Type 2 diabetes	118 (81.9)	193 (73.9)	
Early gestational diabetes	26 (18.1)	68 (26.1)	
Pregnancy characteristics			
Nulliparity (*n*, %)	96 (66.7)	40 (15.3)	<0.001
Gestational age at delivery (weeks) (median, IQR)	37.4 (36.5–38.8)	37.7 (36.6–38.7)	0.933
Induction of labor (*n*, %)	127 (88.2)	197 (75.5)	0.002
Birthweight (grams) (median, IQR)	3198 (2693–3524)	3216 (2880–3586)	0.432

Abbreviations: CD, cesarean delivery; IQR, interquartile range; SD, standard deviation; VD, vaginal delivery.

There were no differences in timing of diabetes diagnosis, metformin use, or neonatal birthweight between groups (Table 1). Compared to those with a VD, participants with an unplanned CD had greater median gestational weight gain (11.6 kg [interquartile range, IQR 6.8–15.4] vs. 10 kg [IQR 4.4–14.9], *p* = 0.019), enrollment HbA1c ≥ 6.5% (69% vs. 56%, *p* = 0.024), third trimester HbA1c ≥ 6.5% (39% vs. 25%, *p* = 0.046), and higher median glucose levels in labor (130.0 mg/dL [IQR 112.0–161.5] vs. 121.0 mg/dL [IQR 100.0–144.0], *p* = 0.003) (Table 2). Of the 99 participants with HbA1c ≥ 6.5% at enrollment, 45% (45/99) had an HbA1c ≥ 6.5% in the third trimester. Overall, there was fair agreement between enrollment HbA1c and third‐trimester HbA1c among participants (kappa 0.34; 95% CI 0.23, 0.46).

**TABLE 2:** Logistic regression analyses evaluating factors associated with unplanned CD.

Exposures^a^	Unplanned CD	VD	Unadjusted OR (95% CI)	Adjusted^b^ OR (95% CI)
	(*n* = 144)	(*n* = 261)		
HbA1c at enrollment (%) (median, IQR)	7.3 (6.2–9.4)	6.85 (5.9–8.5)	1.15 (1.03–1.29)	1.27 (1.10, 1.46)
HbA1c at enrollment ≥ 6.5% (*n*, %)	87 (68.5)	133 (56.4)	1.68 (1.07–2.65)	2.26 (1.28, 3.98)
HbA1c in 3^rd^ trimester ≥ 6.5% (*n*, %)	26 (39.4)	29 (25.2)	1.93 (1.01–3.69)	2.78 (1.21, 6.39)
Gestational weight gain (kg) (median, IQR)	11.6 (6.8–15.4)	10.0 (4.4–14.9)	1.01 (1.00–1.03)	1.01 (0.99, 1.03)
Induction (*n*, %)	127 (88.2)	197 (75.5)	2.43 (1.36–4.33)	2.12 (1.08, 4.17)
Highest glucose level in labor (mg/dL) (median, IQR)	130.0 (112.0–161.5)	121.0 (100.0–144.0)	1.01 (1.00–1.01)	1.01 (1.00, 1.01)

Abbreviations: CD, cesarean delivery; IQR, interquartile range; OR, odds ratio; VD, vaginal delivery.

^a^ Logistic regression was performed to test the association between unplanned CD and each of the six exposures, with adjustment for nulliparity, Hispanic origin, marital status, pre‐existing hypertension requiring medication, preeclampsia, and cigarette use in pregnancy.

^b^ Obtained using logistic regression.

In multivariable adjusted analysis, enrollment HbA1c (mean gestational age 11.2 weeks) was associated with CD (7.3% for CD vs. 6.85% for VD; aOR 1.27 [95% CI 1.10, 1.46]) (Table 2). HbA1c ≥ 6.5% at enrollment (69% for CD vs. 56% for VD; aOR 2.26 [95% CI 1.28, 3.98]), HbA1c ≥ 6.5% in the third trimester (39% for CD vs. 25% for VD; aOR 2.78 [95% CI 1.21, 6.39]), and induction (88% for CD vs. 76% for VD; aOR 2.12 [95% CI 1.08, 4.17]) were associated with unplanned CD. There was no difference in gestational weight gain or the highest glucose level in labor between groups. When limited to nulliparous patients only (*n* = 136), associations remained significant for enrollment HbA1c, HbA1c ≥ 6.5% at enrollment, and induction (see the Supporting Information File).

**FIGURE 2:**
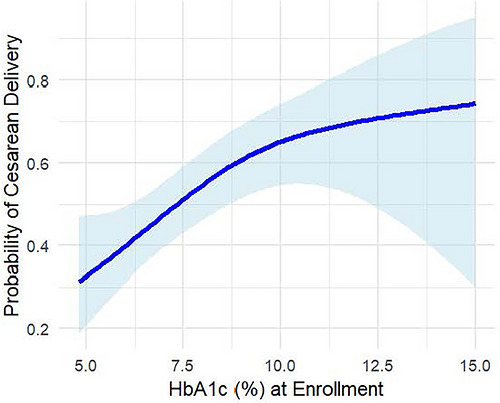
Association between enrollment HbA1c and cesarean delivery (CD). Smoothed probability of CD by HbA1c adjusted for nulliparity, Hispanic ethnicity, marital status, pre‐existing hypertension requiring medication, preeclampsia, and cigarette use in pregnancy. *p* value 0.00371 for the smoothing term, with linear association observed between HbA1c 5% and HbA1c 10%, and leveling off above 10%. HbA1c, hemoglobin A1c.

The smoothed probability of CD by HbA1c at enrollment is depicted in Figure 2. A linear association was observed between HbA1c 5% and HbA1c 10% (*p* = 0.00371 for the smoothing term), with leveling off above 10%.

## 4 COMMENT

### 4.1 Principal finding

The rate of unplanned primary CD among participants with pre‐existing T2D or early GDM was 36%. Elevated early pregnancy HbA1c was independently associated with a significantly increased risk of unplanned primary CD. There was a significant linear relationship between early pregnancy HbA1c and CD for HbA1c values between 5% and 10%. In addition, there was an association between HbA1c ≥ 6.5% in the third trimester and induction of labor with unplanned CD.

### 4.2 Results in the context of what is known

The overall CD rate among MOMPOD study participants was 63%. As noted by Boggess et al. [12], this rate is significantly higher than the national average of 32% [3], but consistent with the rate observed for pregnant individuals with pre‐existing diabetes in prior studies [7, 13]. In our study, the rate of unplanned or emergency primary CD (36%) was also higher than that reported in the general population (approximately 10% among multiparous women and 20%–30% among nulliparous women) [14, 15]. The co‐existence of other maternal risk factors for CD in the general population may contribute to the increased rate of CD observed in this population. Consistent with a prior study, we found an increased risk of unplanned CD among pregnant individuals who were nulliparous and had a hypertensive disorder [6, 14]. We also found a greater risk of unplanned CD among women undergoing induction of labor. While several studies have found that induction of labor does not increase the risk of CD, most notably the 2018 ARRIVE trial [16], it is important to note that many studies excluded patients with pregestational diabetes, who may undergo induction earlier than 39 weeks, particularly in the setting of poor glycemic control. Therefore, findings from such studies may not be generalizable to this high‐risk population. Interestingly, we did not find a significant association between unplanned CD risk and neonatal birthweight. These findings are consistent with those reported by Fischer et al. in a study of 164 pregnant individuals with pre‐existing diabetes undergoing a trial of labor [6]. We suspect that this may be, at least in part, due to the use of planned CD for pregnancies with suspected macrosomia, which were excluded from our analysis.

In our study, the enrollment HbA1c level obtained on average at 11.2 weeks, which reflects glucose levels over the preceding 2 to 3 months, served as an indicator of periconceptional or early pregnancy glycemic control. A higher HbA1c level in early pregnancy was associated with a higher risk for unplanned CD. The leveling off in the CD risk observed at HbA1c levels above 10% may be explained by biological limitations of the effect of HbA1c on the risk of CD beyond 60%–70%. A similar HbA1c inflection point of 9%–10% has been demonstrated in studies of diabetic retinopathy, suggesting a “maximum damage threshold” [17]. Further studies are needed to further refine the magnitude of association between increased CD risk and early pregnancy HbA1c, which is likely clinically significant based on our findings.

First‐trimester HbA1c levels are associated with several immediate and downstream complications. For example, periconception HbA1c levels of greater than 7% are associated with an increased risk of early pregnancy loss, congenital anomalies [1, 18], as well as an increased risk of preterm delivery [19]. Studies in individuals with Type 1 diabetes have shown that those with an HbA1c greater than 6.5% in the first trimester—despite achieving an HbA1c less than 6% in the third trimester—have increased odds of having a large‐for‐gestational age infant compared to those who achieved target HbA1c levels throughout pregnancy [20]. Our study demonstrates an independent association between periconception/early pregnancy HbA1c level and risk of unplanned CD. Although the mechanism by which first‐trimester glucose control impacts mode of delivery remains unclear, our findings suggest that the risk of unplanned CD may persist throughout pregnancy, regardless of neonatal birthweight. This finding further supports existing evidence that early pregnancy HbA1c is a valuable predictor of pregnancy outcomes.

### 4.3 Clinical and research implications

The current focus of preconception counseling for individuals with pre‐existing diabetes is improved glycemic control to reduce the risk of congenital anomalies and optimize maternal health. Our study suggests that improved periconception glycemic levels may also help reduce the risk of unplanned or emergency CD among individuals with pre‐existing diabetes attempting VD. As most pregnant individuals prefer to avoid CD [21], reducing risk of unplanned or emergency CD may represent a tangible motivating factor for individuals with pre‐existing diabetes to optimize their glycemic control prior to pregnancy. Moreover, our study further emphasizes the importance of considering factors beyond estimated birthweight when counseling patients on mode of delivery. Prior studies have demonstrated an association between suboptimal pregnancy glucose control among pregnant people with pregestational diabetes and social determinants of health [22, 23, 24]. Consideration of barriers to optimal glucose control, such as food insecurity, should be considered when caring for and counseling people with pregestational diabetes who are pregnant or planning pregnancy.

This study adds to the existing literature on risk factors for CD among pregnant individuals with diabetes, specifically those with pre‐existing T2D and early GDM. Clinical tools have been developed to predict the risk of CD among patients undergoing a trial of labor after a CD or an induction of labor [25, 26]. Further studies should explore whether CD risk prediction tools for individuals with pre‐existing diabetes could help guide delivery management for this high‐risk population. Preventing potentially unnecessary primary elective CD and reducing unplanned CD among pregnant individuals with diabetes may help decrease maternal morbidity [27]. Standardized tools may help reduce the effect of provider bias on labor management for pregnant individuals with pre‐existing diabetes, which has been proposed as a potential contributor to increased rates of unplanned CD among this patient population [7].

### 4.4 Strengths and limitations

This study has several strengths including a relatively large sample size, utilization of robustly collected data from a large US‐based RCT, and inclusion of a diverse patient population representative of the US population. However, there are certain study conditions that may limit generalizability. First, all MOMPOD participants were treated with insulin, but some pregnant patients with T2D and early GDM can be managed with diet alone or with oral hypoglycemics [28]. Second, the monitoring of trial participants may have led to better adherence to diabetes treatment compared to the real‐world setting where pregnant people may not receive the same level of prenatal care, particularly in the first trimester. Nevertheless, these factors are likely to enhance glucose control among study participants, which would be expected to lower the rate of CD. Therefore, the increased risk of CD among participants with elevated early pregnancy HbA1c in our study may be underestimated compared to the general population, who may not receive insulin treatment and/or have less intensive monitoring for treatment adherence. Third, we could not perform additional analyses according to the indication for cesarean, as multiple indications could exist per patient. Finally, while we found a significant association between HbA1c ≥ 6.5% in the third trimester and unplanned CD, this value was missing for 55% of participants, which was significantly more than for any of the other tested exposures, limiting our ability to draw conclusions regarding the associations between elevated early pregnancy HbA1c and elevated third‐trimester HbA1c, and between elevated third‐trimester HbA1c and unplanned CD.

## 5 CONCLUSIONS

Early pregnancy HbA1c is independently associated with unplanned primary CD in pregnant individuals with T2D or early GDM attempting VD. Preconception counseling should focus on improved glycemic levels prior to and in early pregnancy as a potential strategy to reduce the risk of unplanned CD and associated morbidity for pregnant individuals with pre‐existing diabetes or diabetes diagnosed in early pregnancy.

## CONFLICT OF INTEREST STATEMENT

The authors report no conflicts of interest.

## ETHICS STATEMENT

Institutional Review Board approval was obtained for the parent study and data analysis (protocol number AAAR2412).

## Supporting information



SUPPORTING INFORMATION
