# Supplementary material for: Risk factors for unplanned cesarean delivery among pregnant individuals with pre‐existing diabetes
Source: Pregnancy (Hoboken). 2026 May 20;2(3):e70303. doi: 10.1002/pmf2.70303 (PMC13344378; doi:10.1002/pmf2.70303)
Supplement: Supplementary file 1 [file PMF2-2-e70303-s001.docx]

Supplementary File

| **Exposures** | **Adjusted***  **OR** **(95% CI)** | **Adjusted* for multiple comparisons^**  **OR (95%)** | **Adjusted° OR (95%) among nulliparous participants**  **(n=136)** |
| --- | --- | --- | --- |
| **HbA1c at enrollment** (%)  (median, IQR) | 1.27 (1.10, 1.46) | 1.27 (1.05, 1.54) | 1.62 (1.17, 2.24) |
| **HbA1c at enrollment ≥6.5%**  (n, %) | 2.26 (1.28, 3.98) | 2.26 (1.07, 4.97) | 4.53 (1.95, 10.53) |
| **HbA1c in 3^rd^ trimester ≥6.5%**  (n, %) | 2.78 (1.21, 6.39) | 2.78 (0.92, 8.89) | 7.83 (0.83, 73.53) |
| **Gestational weight gain** (kg) (median, IQR) | 1.01 (0.99, 1.03) | 1.01 (0.90, 1.03) | 1.00 (0.97, 1.03) |
| **Induction** (n, %) | 2.12 (1.08, 4.17) | 2.12 (0.88, 5.51) | 3.72 (1.28, 10.83) |
| **Highest glucose level in labor** (mg/dL) (median, IQR) | 1.01 (1.00, 1.01) | 1.01 (1.00, 1.01) | 1.00 (0.99, 1.01) |

Table A1. Sensitivity Analysis

*Adjusted for nulliparity, marital status, Hispanic ethnicity, preeclampsia, pre-existing hypertension and cigarette smoking

^Using the Bonferroni method adjusting for k=6 comparisons

°Adjusted for marital status, Hispanic ethnicity, preeclampsia, pre-existing hypertension and cigarette smoking
